# Genome-Wide Characterization and Gene Expression Analyses of GATA Transcription Factors in Moso Bamboo (*Phyllostachys edulis*)

**DOI:** 10.3390/ijms21010014

**Published:** 2019-12-18

**Authors:** Taotao Wang, Yong Yang, Shuaitong Lou, Wei Wei, Zhixin Zhao, Yujun Ren, Chentao Lin, Liuyin Ma

**Affiliations:** 1 Basic Forestry and Proteomics Research Center, College of Forestry, Fujian Provincial Key Laboratory of Haixia Applied Plant Systems Biology, Fujian Agriculture and Forestry University, Fuzhou 350002, China; fjnlwtt@163.com (T.W.); yongwithyang@163.com (Y.Y.); 2 Fujian Provincial Key Laboratory of Plant Functional Biology, College of Life Sciences, Fujian Agriculture and Forestry University, Fuzhou 350002, China; Loushuaitong@163.com (S.L.); wwei_0501@163.com (W.W.); ryj@fafu.edu.cn (Y.R.); 3 College of Biopharmaceutical and Food Engineering, Shangluo University, Shangluo 726000, China; zxzhao@slxy.edu.cn; 4 Department of Molecular, Cell and Developmental Biology, University of California, Los Angeles, CA 90095, USA; clin@mcdb.ucla.edu

**Keywords:** GATA genes, transcription factor, gene expression, rhizome development, shoot rapid-growth, moso bamboo

## Abstract

Moso bamboo is well-known for its rapid-growth shoots and widespread rhizomes. However, the regulatory genes of these two processes are largely unexplored. GATA transcription factors regulate many developmental processes, but their roles in moso bamboo height control and rhizome development remains unexplored. Here, thirty-one bamboo GATA factors (PeGATAs) were identified, which are evolutionarily closer to rice than *Arabidopsis*, and their gene expression patterns were analyzed in bamboo development and phytohormone response with bioinformatics and molecular methods. Interestingly, PeGATAs could only be classified into three groups. Phytohormone responsive cis-elements were found in *PeGATA* promoters and the expression profiles showed that *PeGATA* genes might respond to gibberellin acid and abscisic acid but not to auxin at the transcriptional level. Furthermore, *PeGATA* genes have a tissue-specific expression pattern in bamboo rhizomes. Interestingly, most *PeGATA* genes were down-regulated during the rapid-growth of bamboo shoots. In addition, over-expressing one of the *PeGATA* genes, *PeGATA26*, significantly repressed the primary root length and plant height of transgenic *Arabidopsis* plants, which may be achieved by promoting the gibberellin acid turnover. Overall, our results provide insight into the function of GATA transcription factors in bamboo, and into genetic resources for engineering plant height.

## 1. Introduction

Moso bamboo is one of the most abundant non-timber forestry species and provides important resources for food, architecture, papermaking, and fiber [1]. More importantly, moso bamboo is known for its explosive shoot growth rate, with a peak growth rate of 1 m per day [1]. The rapid-growth shoot is largely dependent on the widespread rhizome system, which provides nutrients by absorbing from the soil, and more importantly, transporting from other rhizome-connected adult bamboos [2]. Therefore, studying the development of shoots and rhizomes will help us to understand the rapid-growth regulation of bamboo, and provides effective candidate genes for genetic manipulation of plant height in crop and forestry species.

The GATA factors play important roles in many developmental processes by binding to the consensus DNA sequence (A/T)GATA(A/G) to regulate gene transcription [3,4]. The GATA factors have a highly conserved type IV zinc finger DNA binding domain (CX_2_CX_17–20_CX_2_C), followed by a basic region [5,6,7]. In animals, GATA factors typically contain two zinc finger domains (CX_2_CX_17–20_CX_2_C), while only the C-terminal domain has a DNA binding function [5]. Animal GATAs are involved in development, differentiation, and control of cell proliferation [7]. However, the fungal GATA factors only contain a single zinc finger domain that is highly similar to the C-terminal zinc finger domain of the animal GATA factors [4,8]. In plants, GATA factors contain a CX_2_CX_18_CX_2_C or CX_2_CX_20_CX_2_C zinc finger domain [9,10]. Interestingly, most plant GATA factors have a single zinc finger domain, but very few of them contain two zinc finger domains [9,10,11].

In animals, GATA factors are involved in cell differentiation and organ development. Mutations in animal GATA factors cause severe developmental disorder diseases including anemia, deafness, and renal and cardiac defects [12]. Fungal GATA factors play roles in nitrogen control, siderophore biosynthesis, light-regulated photomorphogenesis, and circadian regulation [4].

Plant GATA factors originate from the identification of GATA motifs in regulatory regions of light and circadian clock responsive genes [13]. The first plant GATA factor was identified from *Nicotiana tabacum* [14]. GATA factors have been identified in many plant species, including *Arabidopsis* (29), rice (28), apple (35), and soybean (64) [9,10,15]. Plant GATA factors are involved in many developmental processes, affecting plant architecture [16], flower development [17], hypocotyl elongation [18], and seed germination [19]. Plant GATA factors mainly regulate nitrogen metabolism [14,20] and plant growth [21,22,23]. They regulate plant growth in two ways: either directly downstream of phytohormone signal transduction [23] or acting as a transcriptional regulator by integrating both light and phytohormone signals [21,22].

Plant GATA factors regulate light signal transduction by combining with the GATA motif in the promoter of light-related genes [24,25]. GATA2 (At2g45050) has also been identified as a key transcriptional regulator of the integration of light and brassinosteroid signaling pathways [22]. Recent evidence suggests that GATA factors are involved in the regulation of plant hormone signal transduction. Two orthologous GATA-type transcription factors—GNC (Gata, nitrate-inducible, carbon metabolism-involved) and GNL (GNC-like) from *Arabidopsis*—were identified as GA-regulated genes [21,23]. Loss-of-function mutants and over-expression lines of *GNC* and *GNL* are functionally related to germination, greening, and flowering time [17]. CHIP (chromatin immunoprecipitation) results show that these two genes are direct targets of PIF transcription factors. *gnc* and *gnl* loss-of-function mutations suppress *ga1* phenotypes, supporting GNC and GNL as important repressors of GA signaling [21]. Another important phytohormone, auxin, is also regulated by GNC and GNL through functioning downstream of ARF2 (Auxin Response Factor 2) [23]. In addition, the GATA factors are induced by cytokinin [26]. These results indicate that GATA factors play crucial roles in plant development and phytohormone-mediated growth. However, the role of GATA factors in rapid-growth and rhizome development remains elusive.

Recently, large-scale transcriptome analysis has shown that light and phytohormones may play important roles in the rapid-growth of bamboo [27,28,29]. In addition, a large number of transcription factor families, involved in the abiotic stress response and flower development, have been studied in moso bamboo [30,31,32]. Although a previous study has functionally characterized the rapid-growth associated gene-*PeGSK1* [1], the rapid-growth associated transcription factor families are largely unexplored in moso bamboo.

In this study, we performed a genome-wide survey of GATA factors in moso bamboo. A total of 31 GATA factors were identified in the moso bamboo genome. The phylogenetic relationship, gene structure and conserved domains of moso bamboo were systematically analyzed. The phytohormone-related cis-elements and gene expression of *PeGATAs* under GA (gibberellin acid), ABA (abscisic acid), and auxin treatment were also characterized. More importantly, the gene expression of *PeGATAs* in different rhizome tissues and rapid-growth shoots were analyzed in detail. In addition, one growth related PeGATA—*PeGATA26*—was over-expressed in *Arabidopsis* to functionally validate its role in regulating plant height. Overall, our results provide information on the involvement of GATA factors in rhizome tissue development and rapid-growth shoots.

## 2. Results

### 2.1. Genome-Wide Characterization of GATA Factors in Moso Bamboo

A total of 31 potential GATA factors were identified in moso bamboo and named *PeGATA1* to *PeGATA31* based on the chromosomal location. The CDS (Coding regions) and protein sequences of *PeGATA* genes are listed in Tables S1 and S2. The detailed information of these PeGATA factors including length of CDS, size of amino acid, molecular weight (MW) of protein, gene location on chromosome, and isoelectric point (PI) are listed in Table 1.

The length of CDS ranged from 366 bp to 1500 bp, and the length of proteins ranged from 122 aa to 499 aa (Table 1). PeGATA29 was the smallest GATA protein with 122 amino acids, and the largest protein was PeGATA6 with 499 amino acids (Table 1). The predicted molecular weight of 31 PeGATA proteins ranged from 13.3 kDa (PeGATA29) to 56.4 kDa (PeGATA6) with an average size of 29.86 kDa (Table 1). The predicted PI of 31 PeGATA factors were all below 10.0, and the minimal protein was PeGATA23 with only 4.75 (Table 1).

### 2.2. Phylogenetic Analysis of Bamboo GATA Factors

**Figure 1:**
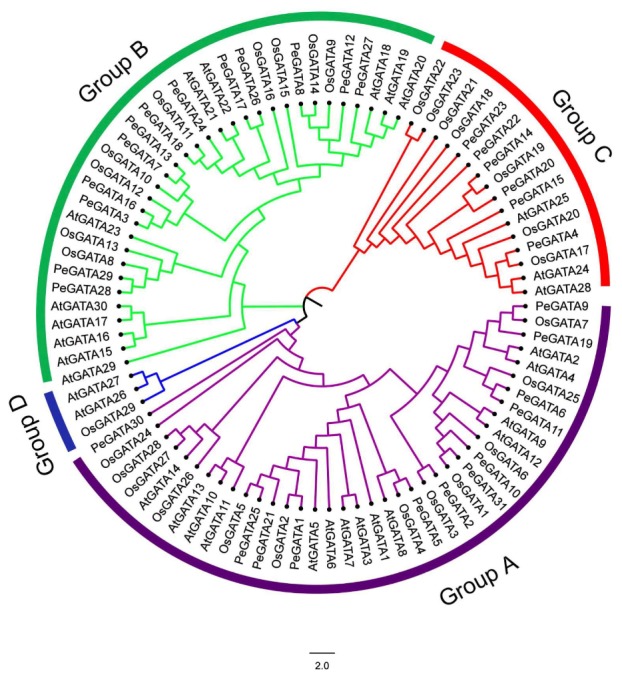
Phylogenetic analysis of GATA factors in bamboo, rice, and *Arabidopsis*. The phylogenetic tree was made based on the amino acid sequences using MEGA7.0 by the neighbor-joining method with 1000 bootstrap replicates. The tree shows four major phylogenetic groups (A to D) indicated by different colors. The gene names of *Arabidopsis*, rice and bamboo GATA factors were begun with “*At*”, “*Os*”, and “*Pe*”, respectively. The exact gene IDs of *Arabidopsis* and rice GATA genes were listed separately in Tables S3 and S4.

Phylogenetic analysis with 90 GATA factor sequences from three species (*Arabidopsis*, rice, and bamboo) showed that these GATA factors were classified into four groups (group A–D, Figure 1). Group A was the largest clade and contained 38 members. In this group, twelve bamboo GATA factors (PeGATA1, -2, -5, -6, -9, -10, -11, -19, -21, -25, -30, and -31) were clustered with 12 rice and 14 *Arabidopsis* GATA factors (Figure 1). Group B formed the second largest clade, containing 33 members, 13 of which were bamboo GATA factors (PeGATA3, -7, -8, -12, -13, -16, -17, -18, -24, -26, -27, -28, and -29), as well as 9 rice and 11 *Arabidopsis* GATA factors (Figure 1). Interestingly, six bamboo (PeGATA4, -14, -15, -20, -22, and -23) and seven rice GATA factors were identified in group C, while only three were identified from *Arabidopsis* (Figure 1). Specifically, it is worth noting that no bamboo GATA factor was found in group D (Figure 1), which indicates that bamboo might lack the functions of group D GATA factors compared with *Arabidopsis* and rice. Consistently, Moso bamboo GATA factors did not cluster with *Brachypodium distachyon* and soybean group D GATA factors, as shown in Figure S1.

### 2.3. The Conservation of GATA Motifs in Bamboo GATA Factors

**Figure 2:**
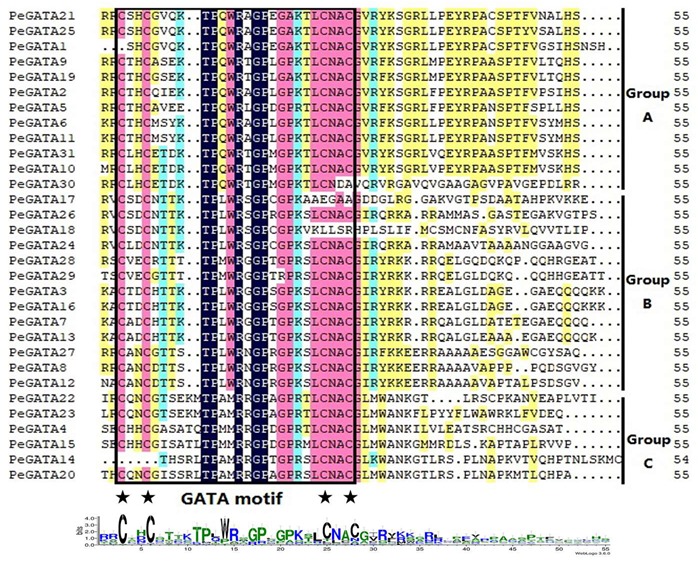
Alignment of the amino acid sequences of bamboo GATA factors. The GATA motif and amino acid positions are marked with a box and an asterisk. The sequence logo of the GATA motif is shown at the bottom.

To further investigate and characterize sequence conservation in the bamboo GATA proteins, multiple sequence alignments were performed using the amino acid sequences of the conserved GATA motifs in 31 PeGATAs (Figure 2). Similar to *Arabidopsis* and rice, moso bamboo did not contain the animal- and fungal-type CX_2_CX_17_CX_2_C zinc finger domains (Figure 2). Twenty-six bamboo GATA factors contained 18 residues in the zinc finger loop (CX_2_CX_18_CX_2_C), while the other five bamboo GATAs had 20 residues in the zinc finger loop (CX_2_CX_20_CX_2_C) (Figure 2). These five bamboo GATA factors were from group C (Figure 2). It has been reported that rice OsGATA24 contains a partial GATA motif [9]. Interestingly, five bamboo GATA factors (PeGATA1, -14, -17, -18, and -30) had a defective GATA zinc finger domain (Figure 2), and the number of partial GATA motif containing factors was larger in bamboo (5) than in rice (1). The results indicated that most of the bamboo GATA factors had a highly conserved GATA motif.

**Figure 3:**
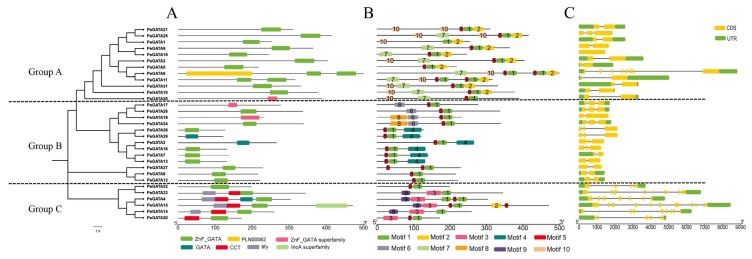
Schematic representation of conserved domains, motifs and gene structure of PeGATA proteins. (**A**) Conserved domains of PeGATA proteins. (**B**) Distribution of conserved motifs in PeGATA proteins identified by the MEME. (**C**) Exon/UTR structures of *PeGATA* genes. Phylogenetic tree construction of the PeGATA proteins based on the amino acid sequences using MEGA7.0. Each color represents a different domain, motif, and structure of the protein. The position of the sequence and the size of exons or UTRs can be estimated by the scale at the bottom.

To further reveal the diversification of GATA factors in moso bamboo, putative conserved functional domains and motifs were also predicted in the NCBI conserved domain database and the MEME (Multiple EM for Motif Elicitation) program. As expected, all of the bamboo GATA factors contain at least one of the GATA motifs (ZnF_GATA, ZnF_GATA superfamily, and GATA, Figure 3A). However, similar to rice but unlike *Arabidopsis*, three bamboo GATA factors contained two zinc finger domains (Figure 3A), while rice OsGATA24 even contains three and a half GATA motifs [9].

Next, through MEME analysis, 10 motifs among the different groups are shown in Figure 3B and the multilevel consensus sequences for these motifs are shown in Table S5. Motif 1 and motif 5 presented in 29 PeGATA proteins and they were annotated as conserved GATA zinc fingers (Figure 3B). However, the other motifs were presented in a group specific manner. For example, three motifs (motif 2, -7, and -10) were exclusively identified in all group A GATA factors except that motif 2 was also identified in PeGATA15 from group C (Figure 3B). Motifs 4, -6, and -8 were only presented in group B GATA factors (Figure 3B). Motif 3 and motif 9 were only predicted from group C GATA factors (Figure 3B). The identification of group-specific motifs from bamboo GATA factors suggests that these motifs might contribute to the functional differences between the bamboo GATA groups.

### 2.4. Gene Structure of Bamboo GATA Genes

The gene structure of the PeGATA factors is shown in Figure 3C and the total exon numbers of *PeGATAs* from each group were calculated. Group A and group B GATA factors contained two or three exons except PeGATA19 (one exon) and PeGATA6 (nine exons) from group A, while group C GATA factors had five to twelve exons (Figure 3C). The gene structure of GATA genes was similar to that of rice [9].

### 2.5. Subcellular Localization of Bamboo GATA Factors

**Figure 4:**
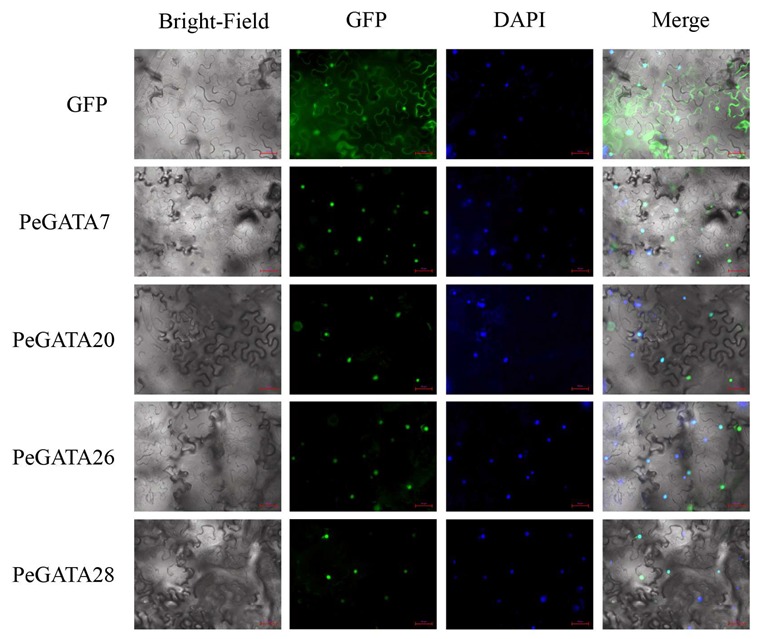
Subcellular localization analyses of bamboo GATA factors. The bamboo GATA genes were cloned and constructed in a modified pCAMBIA3301 vector with a C-terminal GFP fusion. These vectors were transformed into tobacco, and the GFP and DAPI signals were captured from the identical areas by microscopy (20×). The red colored scale-bar represents 50 µm.

Transcription factors are typically located in the nucleus and regulate transcription of the target genes by binding to the cis-elements in their promoters. Consistent with our hypothesis, subcellular localization assays in tobacco showed that bamboo GATA factors (PeGATA7, -20, -26, and -28) were localized in the nucleus according to the GFP and DAPI stain signals (Figure 4). Localization analysis revealed that bamboo GATA factors might localize in the nucleus to regulate gene expression.

### 2.6. The Regulatory Cis-Elements in the Promoters of Bamboo GATA Genes

**Figure 5:**
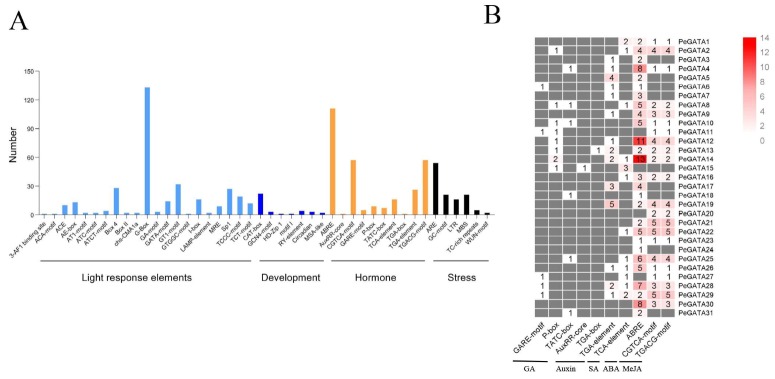
Cis-elements analysis in the promoter of bamboo *GATA* genes. (**A**) Overview of the main types of cis-elements identified from the 1.5-kb upstream sequence of the bamboo *GATA* genes in the PLANTCARE database. (**B**) Hormone-related cis-elements were analyzed and each colored block with numbers represents the number of cis-elements in the bamboo GATA promoter.

To further explore the function and regulatory pattern of the *PeGATA* genes, the PlantCARE database was used to scan the putative cis-elements inside 1500 bp upstream of the translation start site. Light response, development, hormone, and stress-related cis-elements were identified from the promoters of bamboo GATA genes (Figure 5A). Light responsive elements like G-box, GT1, and TCT were widely presented in the promoter of *PeGATA* genes (Figure 5A), and the G-box element has been reported to be involved in the regulation of chlorophyll II biosynthesis in *Arabidopsis* [33].

Next, we further analyzed the hormone responsive elements in bamboo *GATA* promoters. The most dominant hormone responsive element in bamboo *GATAs* is ABRE for recognizing ABA signal and they were identified in the promoters of 27 bamboo GATAs (Figure 5B). The second abundant cis-elements are related to MeJA and 22 bamboo GATAs have them in their promoter regions (Figure 5B). Interestingly, more GA responsive cis-elements were identified in bamboo GATA promoters than auxin-related cis-elements (Figure 5B). Furthermore, the GA-responsive elements were enriched in the group B bamboo GATA factor encoding genes (Figure 5B). Overall, cis-elements analyses indicated that bamboo GATA factors might be involved in response to light and phytohormones to regulate growth.

### 2.7. Gene Expression Analysis of Bamboo GATAs under Exogenous Hormone Treatment

GATA factors are closely related to phytohormones to regulate *Arabidopsis* growth and development [21,23]. Taken together with the identification of phytohormone-related cis-elements in the promoter of the bamboo *GATA* genes (Figure 5B), we rationally hypothesized that the *PeGATA* genes are also tightly regulated by phytohormones. To test our hypothesis, we performed gene expression analysis of the *PeGATA* genes under GA, auxin, and ABA treatment based on the published RNA-seq data or qRT-PCR results [28,34].

**Figure 6:**
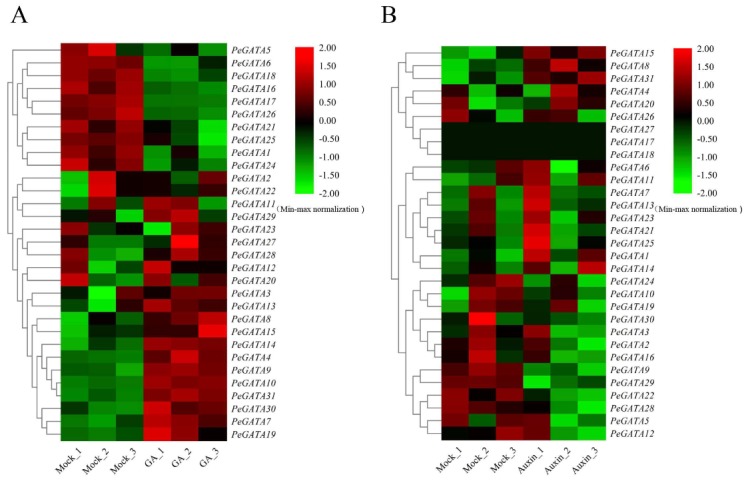
Expression profiles of moso bamboo *GATA* genes under GA and auxin treatments. (**A**) The heatmap of *PeGATAs* gene expression level in moso bamboo seedlings under GA treatment. (**B**) The heatmap of *PeGATAs* gene expression level in moso bamboo seedlings under auxin treatment. The FPKM (Fragments per Kilobase Million) value of *PeGATA* genes were normalized by the min-max method, according to the rows, and presented at the right side. Green represents a low level and red indicates a high level of transcript abundances.

GA acts as one of central regulators in bamboo internode elongation in both seedling and rapid-growth shoot [27,28]. The gene expression analysis of *PeGATA* genes in bamboo seedlings under exogenous GA treatment was performed in a previous study [28]. Briefly, 4-week-old moso bamboo seedlings were treated with or without GA_3_ (100 µM) for 4 h and samples were collected for constructing RNA-seq libraries [28]. The RNA-seq data was mapped with Tophat2 and the expression of bamboo genes was normalized based on fragments per kilobase of transcript per million mapped reads (FPKM) [28]. The normalized expression of *PeGATA* genes was extracted from the published GEOdataset (GSE104596) [28]. A total of 10 *PeGATA* genes (*PeGATA4*, *-8*, *-9*, *-10*, *-14*, *-15*, *-19*, *-30*, and *-31*) showed increased gene expression in GA_3_ (100 µM) treated seedlings compared to that from untreated control (Figure 6A, Table S6). On the contrary, the expression of nine *PeGATA* genes (*PeGATA1*, *-6*, *-16*, *-17*, *-18*, *-21*, *-24*, *-25*, and *-26*) was reduced under GA treatment. *PeGATA26* was the most down-regulated gene with a 56% expression level reduction, followed by *PeGATA18* with a 54% decline. The GA responsive elements were also identified in 10 GA associated bamboo GATA genes (*PeGATA4*, *-6*, *-8*, *-10*, *-14*, *-15*, *-18*, *-19*, *-25*, and *-31*). These results indicate that GA might regulate the gene expression of *PeGATAs*.

Auxin plays important roles in bamboo shoot rapid-growth and seedling development [27,34]. To test the relationship between *PeGATA* gene expression and auxin, we analyzed the gene expression pattern of *PeGATA* genes under NAA treatment (5 µM NAA) in bamboo seedlings, according to the previously published RNA-seq data [34]. Briefly, one-month-old moso bamboo seedlings were treated with or without NAA (5 µM) for 4 h [34]. The roots of these samples were used for an RNA-seq experiment [34], and the RNA-seq data were analyzed exactly the same as the GA experiments [28]. The normalized expression of PeGATA genes was extracted from the GEOdataset (GSE100172). Interestingly, unlike GA treatment, the gene expression of most *PeGATA* genes did not change under auxin treatment (Figure 6B, Table S7). Under auxin treatment, the expression of three *PeGATA* genes (*PeGATA9*, *-29*, and *-28*) was decreased, while the expression of two *PeGATA* genes (*PeGATA8* and *PeGATA15*) was promoted. Moreover, all of these genes except *PeGATA8* contained the auxin responsive cis-elements. Overall, our result suggested that auxin might have a limited effect on the expression regulation of bamboo GATA genes.

**Figure 7:**
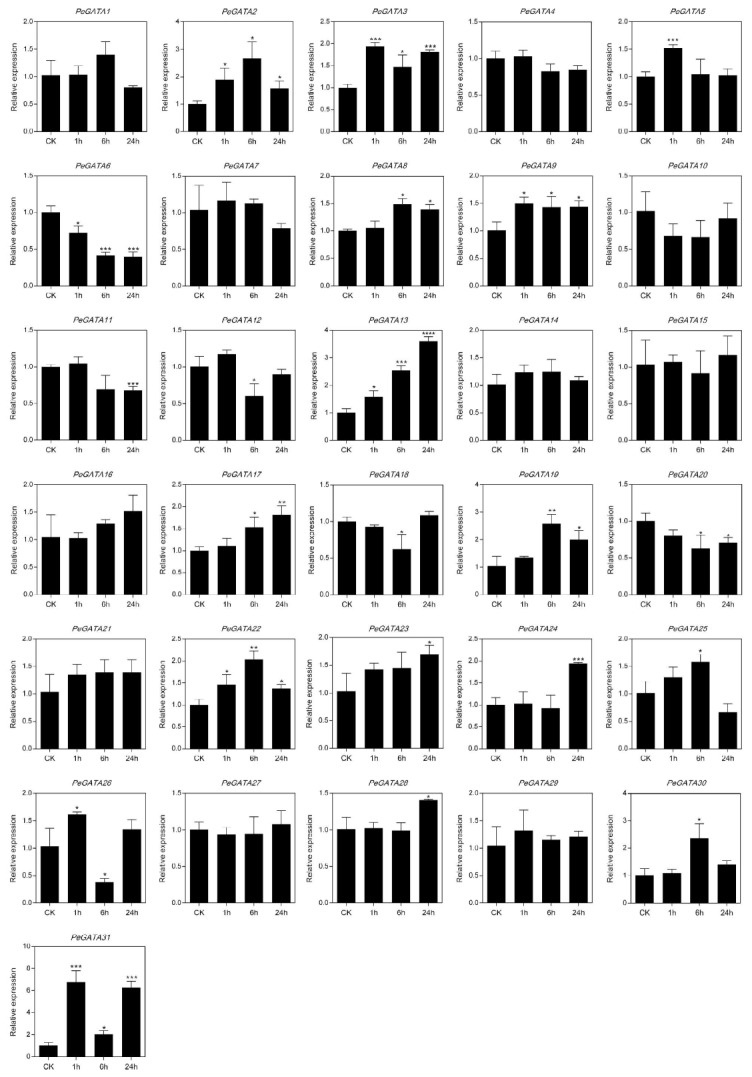
qRT-PCR expression analysis of *PeGATA* genes following abscisic acid (ABA) treatment. Untreated sample was set as control (CK) and its expression levels were normalized to 1. Bars indicate standard deviations (SD) from three biological replicates and asterisks indicate a significant difference between the treatment and CK groups (* *p* < 0.05, ** *p* < 0.01, *** *p* < 0.001).

To test the relationship between *PeGATA* gene expression and ABA, we analyzed the gene expression pattern of all 31 *PeGATA* genes under a time-course ABA treatment (control, 1 h, 6 h, and 24 h) in bamboo seedlings by qRT-PCR. Twenty-one of them (*PeGATA2*, *-3*, *-5*, *-6*, *-8*, *-9*, *-11*, *-12*, *-13*, *-17*, *-18*, *-19*, *-20*, *-22*, *-23*, *-24*, *-25*, *-26*, *-28*, *-30*, and *-31*, Figure 7) changed their expression pattern in one of the ABA time-course treatment stages (Figure 7). ABA treatment increased the expression of fifteen bamboo *GATA* genes (*PeGATA2*, *-3*, *-5*, *-8*, *-9*, *-13*, *-17*, *-19*, *-22*, *-23*, *-24*, *-25*, *-28*, *-30*, and -*31*, Figure 7). Especially, the expression of *PeGATA31* was highly induced by ABA (Figure 7). Interestingly, only five tested bamboo GATA genes (*PeGATA6*, *-11*, *-12*, *-18*, and *-20*) showed reduced expression under ABA treatment (Figure 7). Among the 21 ABA-related bamboo GATA genes, all of them, except *PeGATA11* and *PeGATA22*, contain the ABA responsive cis-elements in their promoters. Overall, our results suggested that ABA might induce the expression of bamboo *GATA* genes.

### 2.8. Gene Expression Analysis of Bamboo GATA Genes during the Rapid-Growth of Bamboo Shoots

**Figure 8:**
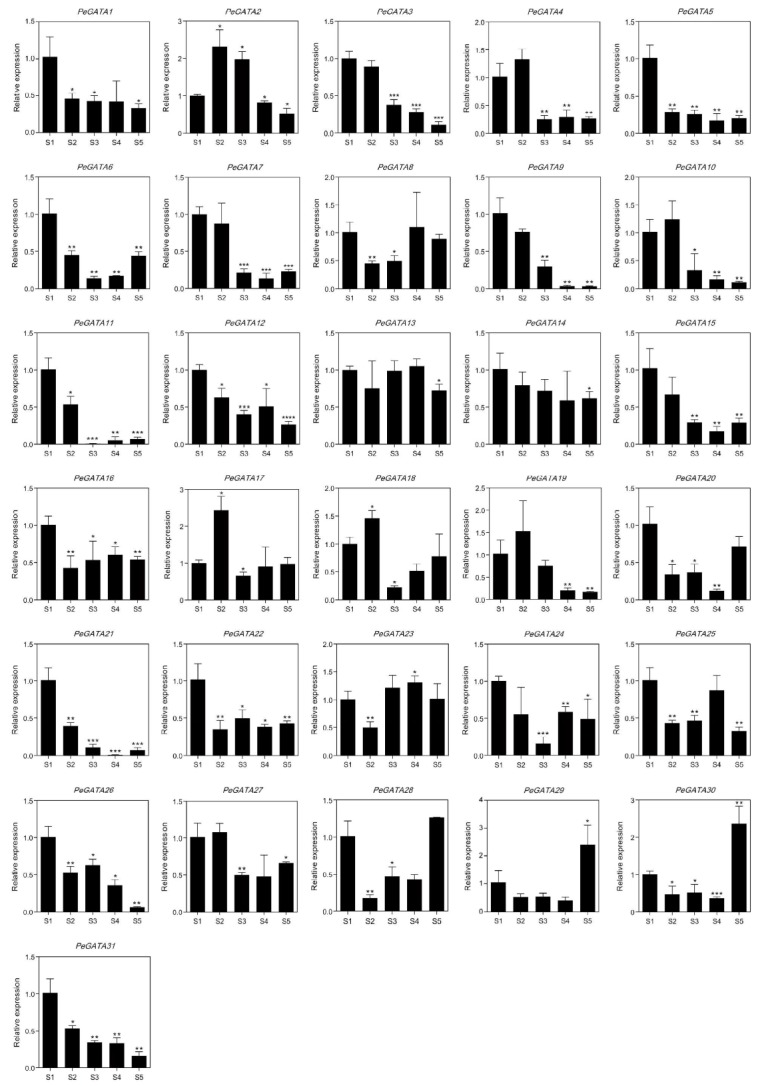
qRT-PCR expression analysis of *PeGATA* genes in bamboo shoots. The *y*-axis and *x*-axis indicate relative expression level at different heights of shoots. S1: 0.15 m shoots; S2: 0.5 m shoots; S3: 1.6 m shoots; S4: 4.2 m shoots; S5: 9 m shoots. Asterisks indicate a significant difference between the higher shoots and the 0.15 m shoots (* *p* < 0.05, ** *p* < 0.01, *** *p* < 0.001).

As the rapid-growth of bamboo shoots is largely determined by phytohormone and nutrients [2,27], and we have demonstrated that *PeGATAs* were differentially expressed under different phytohormone treatments (Figure 6 and Figure 7), we hypothesized that *PeGATAs* might also involve in the fast-growth of bamboo shoots. To validate this hypothesis, the expression profiles of the *PeGATAs* in the fast-growing bamboo shoots (0.15, 0.5, 1.6, 4.2 and 9 m) were examined by qRT-PCR (Figure 8). Interestingly, most of *PeGATA* genes had a lower expression during the rapid-growth of bamboo shoots (Figure 8). Especially, nine *PeGATA* genes decreased their expression of rapid-growth of bamboo (*PeGATA5*, *-6*, *-11*, *-12*, *-16*, *-21*, *-22*, *-26*, and *-31*, Figure 8). Five of them (*PeGATA6*, *-16*, *-21*, *-26*, and *-31*) were responsive to GA treatment, and all of them, except *PeGATA31*, also decreased their expression under GA treatment (Figure 6A). The results indicated that these genes might negatively correlate with the rapid-growth of shoots probably via GA signaling pathway. Other sets of *PeGATA* genes (*PeGATA3*, *-4*, *-7*, *-9*, *-10*, *-15*, and *-24*) did not have change in expression at the 0.5 m shoot developmental stage, but reduced expression at other rapid-growth stages (Figure 8). Five of them were in response to GA treatment and all of them increased expression under GA treatment except *PeGATA24* (Figure 6A). The results suggest that these genes might have function at the 0.5 m shoot development stage and GA might also regulate their expression. In addition, three *PeGATA* genes (*PeGATA2*, *-17*, and *-18*) have increased their expression at the 0.5 m developmental stage. Interestingly, we did not find that any *PeGATA* genes continued to increase their expression along with bamboo shoot development. Overall, our results indicated that *PeGATAs* genes might be involved in the rapid-growth regulation of bamboo shoots.

### 2.9. Gene Expression Analysis of Bamboo GATAs during Rhizome Development

**Figure 9:**
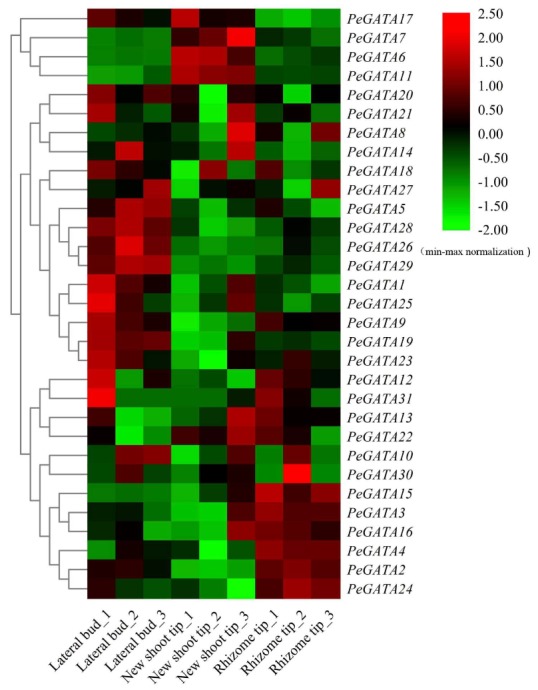
The expression profiles of *PeGATA* genes in different rhizome tissues. Expression values were normalized and presented at the right side, green represents a low level and red indicates a high level of transcript abundances. Each tissue has three replicates.

The lateral buds, rhizome tips, and new shoot tips are important for rhizome development, and the picture of these tissues has been depicted in previous study [2]. The widespread rhizome system is essential for rapid-growth of bamboo shoots through adopting and utilizing nutrients [35]. By analyzing the RNA-seq data from previous study [2], the expression pattern of *PeGATA* genes showed a tissue-specific pattern in rhizome (Figure 9, Table S8).

As shown in Figure 9, thirteen bamboo *GATA* genes might play important roles in the development of different rhizome tissues. *PeGATA17* exclusively reduced expression in rhizome tips, while five *PeGATAs* (*PeGATA2*, *-3*, *-4*, *-15*, and *-24*) increased expression more in rhizome tips than in the other two tissues (Figure 9). More importantly, among them, four *PeGATAs* (*PeGATA4*, *-15*, *-17*, and *-24*) were responsive to GA and all of them, except *PeGATA24*, increased expression under GA treatment (Figure 9). Therefore, the results suggest that these *PeGATA* genes might play important roles in controlling the development of rhizome tips and the phytohormone GA might also be involved in the regulation expression of these genes. Next, the expression of three *PeGATA* genes (*PeGATA6*, *-7*, and *-11*) were exclusively increased in new shoot tips of the bamboo rhizome, and only *PeGATA6* had an opposite expression pattern, from GA treatment. The results suggest that these three *PeGATA* genes might play central roles in new shoot development. Finally, eight *PeGATA* genes (*PeGATA1*, *-9*, *-19*, *-20*, *-23*, *-26*, *-28*, and *-29*) were identified to have an increased expression in the lateral bud compared to the other tissues (Figure 9). Interestingly, three of them (*PeGATA9*, *-28*, and *-29*) were responsive to auxin treatment (Figure 6B). More importantly, all of them have an opposite expression pattern between the lateral bud and auxin treatment (Figure 6B). Therefore, the result indicates that these four GATA genes might be involved in the growth regulation of lateral buds and probably via the auxin pathway.

### 2.10. PeGATA26 May Negatively Regulate Plant Height in Arabidopsis

To understand the function of PeGATA factors, PeGATA26 was chosen as an example to verify the role of PeGATA factors in plant growth. GA, ABA, and MeJA-related cis-elements were also identified in the *PeGATA26* promoter (Figure 5). *PeGATA26* showed higher gene expression in the growth-inactive lateral buds than the growth-active rhizome tips and new shoot tips (Figure 9). Moreover, *PeGATA26* have lower gene expression under GA treatment (Figure 6A), and its expression decreased in rapid-growth bamboo shoots (Figure 8). These results suggest that *PeGATA26* might act as a negative regulator of plant growth and height in moso bamboo. Therefore, we hypothesized that *PeGATA26* plays a crucial role in regulating plant growth. In a previous study, we successfully characterized one of the fast growth-suppressing genes *PeGSK1* by over-expressing it in *Arabidopsis* [1]. Therefore, we used a similar strategy to verify the function of *PeGATA26* in regulating plant growth by over-expressing it in *Arabidopsis*.

**Figure 10:**
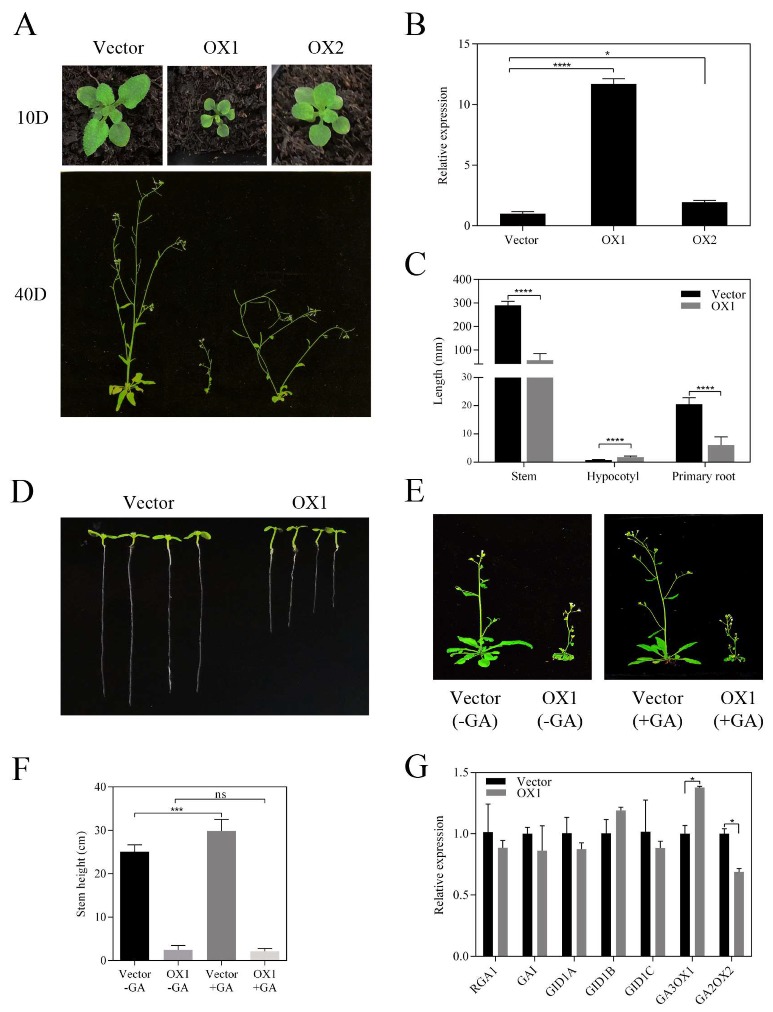
Ectopic expression of *PeGATA26* inhibits the plant height of *Arabidopsis*. (**A**) Over-expression of *PeGATA26* resulted in a dwarf phenotype in *Arabidopsis*. 10D: 10-day-old seedlings, 40D: 40-day-old seedlings. (**B**) The expression level of *PeGATA26* was detected in both transgenic lines with *ACTIN2* gene as internal control. (**C**) Phenotypic analysis of plant height, hypocotyl length, and primary root length in *PeGATA26*-ox1 compared with the control (>30 individual plants). (**D**) Hypocotyl and primary root phenotype of 7-day-old *PeGATA26* over-expression line 1 (*PeGATA26*-ox1). Phenotypic (**E**) and statistical (**F**) analyses of *PeGATA26*-ox1 were resistant to exogenous GA treatment. (**G**) The expression of GA signaling genes was detected by qRT-PCR, and *ACTIN2* was used as an internal control. Asterisks (Student’s t test, * *p* < 0.05, *** *p* < 0.001, **** *p* < 0.0001, ns: no significance, *p* ≥ 0.05) marked significant difference between *PeGATA26*-ox and control.

The homozygous T3 transgenic lines were used to analyze the phenotype, and over-expression of *PeGATA26* resulted in a significant growth retardation phenotype (Figure 10A). Gene expression of *PeGATA26* was successfully detected by qRT-PCR (Figure 10B). The phenotypes between the two over-expressing lines were similar and the intensity of phenotype correlated with the expression of each of the transgenic lines (Figure 10A). Therefore, *PeGATA26* over-expressing line 1 (*PeGATA26*-ox1), with a stronger phenotype, was used for further phenotypic analysis. Interestingly, *PeGATA26*-ox1 showed a significant dwarf phenotype with a dramatic shorter inflorescence compared to the control (Figure 10C), indicating that *PeGATA26* inhibits growth of plant height. Moreover, *PeGATA26* also repressed primary root growth (Figure 10C–D). However, *PeGATA26* promoted *Arabidopsis* hypocotyl length (Figure 10C–D). These results indicate that *PeGATA26* may regulate plant growth in a tissue-specific manner—repressing cell growth in roots and inflorescences, while promoting cell growth in hypocotyls.

As *PeGATA26* was down-regulated under the GA treatment in bamboo seedlings (Figure 6A), we subsequently analyzed whether *PeGATA26* is also regulated by GA in *Arabidopsis*. Interestingly, exogenous GA treatment did not complement the dwarf phenotype of *PeGATA26*-ox1 (Figure 10E–F), indicating that PeGATA26 might act downstream of the GA biosynthesis pathway to regulate plant height. Furthermore, qRT-PCR analyses showed that the expression of GA signaling related genes including *RGA1*, *GAI*, *GID*, *GID1B*, and *GID1C* did not change in the *PeGATA26*-ox1 transgenic line. Instead, the GA turnover-related genes *GA3 oxidase 1* and *GA2 oxidase 2* were significantly increased and reduced, respectively, in the *PeGATA26*-ox1 transgenic line (Figure 10G). The increased *GA3 oxidase 1* and decreased *GA2 oxidase 2* expression is correlated with the promoting GA turnover and thus decreases the GA level [21]. Therefore, PeGATA26 might repress *Arabidopsis* growth via stimulating the GA turnover. Overall, these results suggest that PeGATA26 inhibits the plant root and stem growth in *Arabidopsis*, and—together with the gene expression—might negatively correlate with the growth of shoots in moso bamboo. We concluded that PeGATA26 might be a negative growth regulator for plant height control from *Arabidopsis* to moso bamboo, probably via promoting GA turnover.

## 3. Discussion

Moso bamboo is one of the important non-timber forestry species with great value in providing food and building materials [36]. Moreover, bamboo is known for its fast-growing shoots and widespread rhizomes [2]. It has been reported that several gene families are involved in flower development and abiotic stress [30,31,32,37]; however, the rapid-growth associated transcription factors remain elusive. The genome sequences of moso bamboo [36] and transcriptome studies [2,27,28,34] provide important platforms for the identification of rapid-growth shoot and rhizome development associated gene families. The rapid-growth related genes could provide useful information for genetic manipulation of plant height in the future.

Bioinformatics analysis showed that there are 31 PeGATA factors in moso bamboo (Table 1). The number of bamboo GATA factors is closer to other species, including *Arabidopsis* (29), rice (28), and apples (35) [9,10,15]. Furthermore, most PeGATA factors have a conserved single CX_2_CX_18–20_CX_2_C zinc finger domain that is highly similar to that from *Arabidopsis* and rice (Figure 2) [9]. In addition, the groups of A to C from moso bamboo showed a highly evolutionary conservation compared to *Arabidopsis* and rice (Figure 1). These results indicate that most of the GATA factors from moso bamboo are conserved compared to other species. However, unlike containing only one zinc finger domain in group A, GATA factors from *Arabidopsis* and rice [9], PeGATA6 and PeGATA11 from the bamboo GATA group A, have two GATA-type zinc finger domains (Figure 3A).

Moreover, more protein domains from the bamboo GATA group C were identified compared to that from *Arabidopsis* and rice (Figure 3). Interestingly, a unique feature of the PeGATA factors is that they only have three groups compared to the four groups of *Arabidopsis* and rice (Figure 1). These differences suggest that PeGATAs do have certain specificity compared to that from *Arabidopsis* and rice. Future analysis of the functions of GATA factors, including AtGATA26, AtGATA27, and OsGATA30 from group D (Figure 1), can help us reveal why bamboo lacks these GATA factors.

The first GATA factor is identified according to the light and circadian clock-related cis-elements in its promoters [13]. Moreover, the *Arabidopsis* GATA factors AtGATA1, AtGATA2, and AtGATA4 have been reported to be involved in light regulation of gene expression and photomorphogenesis [22,38]. Thus, the function of the GATA factors can be predicted based on the identification of *cis*-elements from their promoter. In this study, we found that the promoter of *PeGATAs* has many important cis-elements, including light responsive elements, and cell cycle regulation and phytohormone responsive elements (Figure 5), which are closely related to the regulation of plant growth. Thus, *PeGATAs* may be involved in regulating plant growth through these cis-elements to affect their gene expression and further downstream genes.

Bamboo has a well-established rhizome system to develop new shoot tips and widespread rhizome tips [2,35]. However, the lateral buds of bamboo rhizomes are not active and dominant in growth [2]. Therefore, identification of GATAs with different expression patterns in these tissues will help us clarify the role of GATA factors in rhizome development, which presently remains unclear. In this study, we found that eight *PeGATA* genes are highly expressed in lateral buds (Figure 9). Among them, the orthologous gene of *PeGATA9*, *AtGATA2* has been reported to have a function to restrict cell division in the proliferation domain of *Arabidopsis* root meristem [39], and high expression of *PeGATA9* in lateral buds indicates that *PeGATA9* may also involve in inhibiting the cell division in bamboo lateral buds (Figure 9). In contrast, the lower expression of *PeGATA9* in the actively growing new shoot tips and rhizome tips (Figure 9), suggests a negative correlation between *PeGATA9* and cell growth in bamboo rhizome.

Researchers reported that *AtGATA22*, an orthologous gene of *PeGATA18*, is involved in response to cytokinin and negatively regulates root growth in *Arabidopsis* [40]. We found that *PeGATA18* has higher gene expression in lateral buds (Figure 9), suggesting that *PeGATA18* may play a role in negative regulation of cell growth. Overall, these results suggest that these *PeGATAs* may contribute to negatively regulating cell growth in lateral buds. Next, five *PeGATAs* were highly expressed in the new shoot tips compared to in the other two tissues (Figure 9). Furthermore, five *PeGATAs* were highly expressed in the rhizome tips, indicating that they are involved in the growth of the rhizome tips (Figure 9). Once the transformation system is ready in the future or by mutagenesis with EMS, functional characterization of these *PeGATAs* in moso bamboo will help us elucidate the exact role of PeGATAs in rhizome growth control.

The correlation between GATA factors and GA or auxin has been extensively studied in *Arabidopsis* [21,23,39,41]. In this study, we found that the gene expression of 19 *PeGATAs* changed under GA treatment, while only five *PeGATAs* responded to auxin treatment (Figure 6). In addition, motif analysis indicated that the promoter of *PeGATAs* has more GA-related cis-elements than auxin (Figure 5B). Our results indicate that GA, rather than auxin, frequently regulates the expression of *PeGATAs* in moso bamboo.

Gene expression analysis showed that most *PeGATAs* change their expression during the rapid-growth of bamboo shoots (Figure 8). For example, the expression of *PeGATA9* was down-regulated over 30 times in the late rapid-growth stage (9 m) than the early stage (0.15 m) (Figure 8). The results indicate that *PeGATA9* may negatively regulate the rapid-growth of bamboo shoots. Identification of many rapid-growth-related *PeGATAs* indicates that *PeGATAs* are involved in regulating the bamboo shoot. The rapid-growth of bamboo shoots is tightly controlled by phytohormones [27]. Current studies reveal that ABA is the only negative regulator of fast-growing shoots, while BR, auxin, GA, and cytokinin antagonize with ABA to promote rapid-growth of bamboo shoots [27]. Interestingly, all of the 19 GA-related and 21 ABA-related *PeGATAs* showed differential expression in at least one of the rapid-growth stages (Figure 6B and Figure 8), suggesting that GA and ABA may regulate rapid-growth of bamboo shoots via modulating the gene expression of *PeGATAs*.

To understand the function of GA-related *PeGATAs* in plant height control, *PeGATA26* was selected to validate its role in *Arabidopsis* growth (Figure 10). The orthologous gene of *PeGATA26* in *Arabidopsis* is *GNC* (*AT5G56860*), which plays negative roles in seed germination, flowering, and leaf elongation growth [21]. Over-expressing *GNC* repressed *Arabidopsis* germination, leaf expansion, and flowering [21]. Similarly, over-expression of *PeGATA26* in *Arabidopsis* resulted in growth retardation phenotypes such as dwarfism and shorter primary root length, and the *PeGATAs* over-expressed lines were resistant to GA treatment (Figure 10). Future study focused on complementing the *PeGATA26* into *Arabidopsis gnc* mutants will help us clarify the functional similarity and difference between *PeGATA26* and *GNC*. Overall, these results further support that *PeGATAs* could regulate plant heights from *Arabidopsis* to moso bamboo via downstream of the GA pathway.

## 4. Material and Methods

### 4.1. Plant Materials and ABA Treatment

The shoots of moso bamboo (*Phyllostachys edulis*, 0.15 m, 0.5 m, 1.6 m, 4.2 m, and 9 m) used in this study were collected from wild bamboo forestry with no permission needed in JianOu County (E 118°28′; N 27°00′), Fujian Province, China. The moso bamboo plants were authenticated by Zhenguo Xu (Guangxi Forestry Research Institute, Nanning, Guangxi Province, China) and a voucher specimen has been deposited in Herbarium, Guangxi Forestry Research Institute (voucher No. 2018060325). To ensure the high similarity growth conditions of bamboo shoots, all the shoots were collected in an area of 50 × 50 m^2^ [42]. The middle internode of different heights of bamboo shoots, which was determined using an equal division method, were sampled and stored in liquid nitrogen immediately, as recommend by a previous study [43]. For ABA treatment, 4-week-old moso bamboo seedlings grown in soil were transformed to Hoagland solution for an additional one week of cultivation and then treated with 10 µM ABA (CAISSON, A036-100MG, Smithfield, UT, USA) in a time-course experiment (0, 1, 6, and 24 h). Samples were harvested and RNA was isolated to perform future expression analysis.

### 4.2. Identification of GATA Factors in Moso Bamboo

To identify the GATA factors, the genome and protein sequences of moso bamboo were downloaded from BambooGDB database (http://forestry.fafu.edu.cn/db/PhePacBio/download.php) [36]. GATA protein sequences from *Arabidopsis* and rice were obtained from previous published data [9]. We performed multiple sequence BLAST and alignment with an expected value of 10. The HMMER profile of the GATA domain (PF00320) from Pfam (http://pfam.xfam.org/) was used to search the bamboo protein database with a threshold: *e*-values < 10^−5^ [44]. The bamboo GATA factors were determined with the criteria: Present in both BLAST and HMMER motif analysis lists. The number of amino acid, molecular weights (MWs), and isoelectric points (PI) of bamboo GATA factors were predicted by ProtParam (https://web.expasy.org/protparam/).

### 4.3. Phylogenetic Tree, Conserved Domain, Motif Recognition and Cis-Elements Analyses

Multi-sequence alignment of the GATA protein sequences was carried out by ClustalX [45], and phylogenetic tree was constructed using MEGA7 by the Neighbor-Joining method (bootstrap analysis for 1000 replicates) [46]. Conserved domains were obtained from NCBI (https://www.ncbi.nlm.nih.gov/cdd) [47] and motifs were analyzed using MEME with default parameters (version 5.0.5, http://meme-suite.org/tools/meme) [48]. For cis-elements analysis, DNA sequences from 1.5 kb upstream region of each *PeGATA* gene were used to scan any potential cis-element using the PlantCARE database (http://bioinformatics.psb.ugent.be/webtools/plantcare/html/) [49].

### 4.4. Subcellular Localization Analysis

To verify the location of PeGATAs, the full-length CDSs without stop codon from four *PeGATA* genes were cloned into a modified pCAMBIA3301 vector with C-terminal GFP, as described in a previous study [1]. The *ACTIN2::PeGATAs*-GFP and the *ACTIN2*::GFP control constructs were then transiently transformed into tobacco as described in a previous study [50]. We used 1 mg/L DAPI (SIGMA, D9542, St. Louis, MO, USA) to stain the nucleus for 30 min, and the identical region was observed using a microscope (20×, Zeiss, Axio Observer A1, Jena, Germany) to detect GFP and DAPI fluorescence signals. The two photographs with identical regions from GFP and DAPI were then merged into a new photograph, in which the overlapping of GFP and DAPI signals represented a nuclear localization of bamboo GATA factors.

### 4.5. Gene Expression Analysis

To investigate gene expression levels of the *PeGATA* genes in different tissues or hormone treatments, RNA-seq data was downloaded from the Short Read Archive (SRA) database for the lateral buds, rhizome tips, and new shoot tips (SRP093919) [2], and bamboo seedlings under GA and auxin treatment (GSE104596 and GSE100172) [28,34], respectively. The pair-end reads were mapped to the moso bamboo reference genome using Tophat2, and differentially expressed genes were detected by Cufflinks with default parameters [51].

qRT-PCR analysis was performed for each member of the GATA family genes during the rapid-growth of bamboo shoots or ABA treated seedlings. Total RNA was extracted from the bamboo samples using the HiPure Plant RNA Mini Kit (Magen, R4151-02, Guangzhou, China) and 1 μg RNA was taken for reverse transcription into cDNA using a commercial Kit (Monad, RN05004M, Suzhou, China). Primers for qRT-PCR were designed on Primer3 (http://primer3.ut.ee/) using the CDS of each *PeGATA* gene. qRT-PCR were performed using MonAmp™ ChemoHS qPCR Mix (Monad, RN04002M, Suzhou, China) in a 20 µL reaction. The following program was used for qRT-PCR: 95 °C for 5 min; 40 cycles of 95 °C for 10 s, 60 °C for 10 s, and 72 °C for 30 s. For qRT-PCR data analyses under ABA treatment, the expression of *PeGATA* genes in treated bamboo seedlings was normalized to control condition. For qRT-PCR data analyses for rapid-growth bamboo shoots, the expression of *PeGATA* genes from the S2–S5 stages was normalized to the expression of the S1 stage. The expression of GA signaling genes in *PeGATA26* transgenic lines was normalized according to the expression in empty vector over-expressing control lines.

### 4.6. Ectopic Expression Analysis

*PeGATA26* was cloned and expressed in *Arabidopsis* following exactly the procedures for the *PeGSK1* in previous study [1]. The T3 generation seedlings were used for phenotype analysis. Seven-day-old *PeGATA26* over-expressing transgenic or empty vector control lines (>30 individual plants) were used to measure the primary root length or hypocotyl length with ImageJ [1], respectively. The height of 40-day-old *Arabidopsis* plants was measured using a ruler. The *p*-value was calculated by T-test and the number of asterisks represented the extent of significance difference (* *p* < 0.05, ** *p* < 0.01, *** *p* < 0.001, **** *p* < 0.0001).

The primers used in this study are listed in Table S9.

## 5. Conclusions

With the explosive growth rates of bamboo shoots and widespread rhizomes, the identification of key regulatory genes in the bamboo shoot and rhizome growth control will provide important genetic resources for the genetic manipulation of plant height. In this study, we characterized 31 GATA factors from moso bamboo. More importantly, the gene expression of *PeGATAs* is closely related to the development of rhizome tissues and the rapid-growth of bamboo shoots. Moreover, the gene expression of *PeGATAs* might be regulated by the phytohormone-GA and ABA in bamboo. In addition, functional characterization of *PeGATA26* in *Arabidopsis* provides insight into how *PeGATAs* might regulate plant height from *Arabidopsis* to bamboo via downstream of the GA signaling pathway. In summary, our results provide evidence that the GATA transcription factor might regulate the development of rhizome tissues and the rapid-growth of bamboo shoots.

## Figures and Tables

**Table 1:** GATA factors in moso bamboo.

Name	Gene ID	Location	ORF Length (bp)	Size (aa)	MW (kDa)	PI	Group
*PeGATA1*	PH01000001G0820	603239–605821 (− strand)	762	253	27.2	7.11	A
*PeGATA2*	PH01000036G1110	651571–655167 (+ strand)	1212	403	42.4	5.2	A
*PeGATA3*	PH01000040G1560	1013911–1015300 (− strand)	801	266	28.7	9.77	B
*PeGATA4*	PH01000114G0660	460195–464981 (+ strand)	912	303	32.2	5.96	C
*PeGATA5*	PH01000157G0800	521887–523791 (− strand)	654	217	23.2	6.15	A
*PeGATA6*	PH01000162G1360	945504–954318 (+ strand)	1500	499	56.4	9.4	A
*PeGATA7*	PH01000232G0180	85809–87165 (− strand)	420	139	15.6	9.23	B
*PeGATA8*	PH01000242G0460	296415–297844 (− strand)	648	215	22.6	8.2	B
*PeGATA9*	PH01000263G0760	473691–475362 (− strand)	1095	364	37.5	7.72	A
*PeGATA10*	PH01000284G0590	365850–367843 (− strand)	1131	376	39.3	5.89	A
*PeGATA11*	PH01000417G1130	669097–674119 (− strand)	948	315	35.8	8.87	A
*PeGATA12*	PH01000468G1050	681872–683301 (+ strand)	663	220	22.7	5.78	A
*PeGATA13*	PH01000604G0620	351426–352613 (+ strand)	399	132	14.6	9.36	A
*PeGATA14*	PH01000750G0690	435897–442169 (+ strand)	777	258	28.1	8.18	C
*PeGATA15*	PH01000836G0660	444348–452795 (− strand)	1413	470	51.6	8.57	C
*PeGATA16*	PH01000985G0260	141878–143100 (+ strand)	402	133	14.8	9.87	B
*PeGATA17*	PH01001002G0190	175975–177694 (+ strand)	831	276	29.4	8.98	B
*PeGATA18*	PH01001129G0380	296297–297943 (+ strand)	699	232	26	9.16	B
*PeGATA19*	PH01001155G0480	343290–344746 (− strand)	741	246	25.3	9.66	A
*PeGATA20*	PH01001253G0390	263024–267886 (− strand)	516	171	18.9	9.95	C
*PeGATA21*	PH01001451G0450	270274–272851 (− strand)	930	309	32.6	8.68	A
*PeGATA22*	PH01001557G0370	244289–248001 (+ strand)	594	197	20.7	9.14	C
*PeGATA23*	PH01001584G0350	297120–303918 (− strand)	1035	344	37.8	4.75	C
*PeGATA24*	PH01001907G0160	109783–111580 (+ strand)	1017	338	36.2	9.26	B
*PeGATA25*	PH01002105G0190	151795–153666 (+ strand)	1248	415	44	8.6	A
*PeGATA26*	PH01002473G0050	19960–21655 (− strand)	1011	336	36.1	9.64	B
*PeGATA27*	PH01002681G0110	59436–60698 (− strand)	690	229	24	8.49	B
*PeGATA28*	PH01002830G0260	174984–177126 (− strand)	381	126	13.8	9.69	B
*PeGATA29*	PH01003365G0100	53196–55342 (+ strand)	369	122	13.3	9.4	B
*PeGATA30*	PH01003433G0110	88789–92096 (− strand)	1167	388	39.8	9.33	A
*PeGATA31*	PH01004789G0060	58264–61570 (− strand)	993	330	35	6.06	A

bp: base pair, aa: amino acids, MW: molecular weight, PI: isoelectric point, kDa: kilodalton, ORF: open reading frame.

## Supplementary Materials

Supplementary materials can be found at https://www.mdpi.com/1422-0067/21/1/14/s1.
