# Supplementary figures and images for: Genome-Wide Characterization and Gene Expression Analyses of GATA Transcription Factors in Moso Bamboo (Phyllostachys edulis)
Source: Int J Mol Sci. 2019 Dec 18;21(1):14. doi: 10.3390/ijms21010014 (PMC6982067; doi:10.3390/ijms21010014)

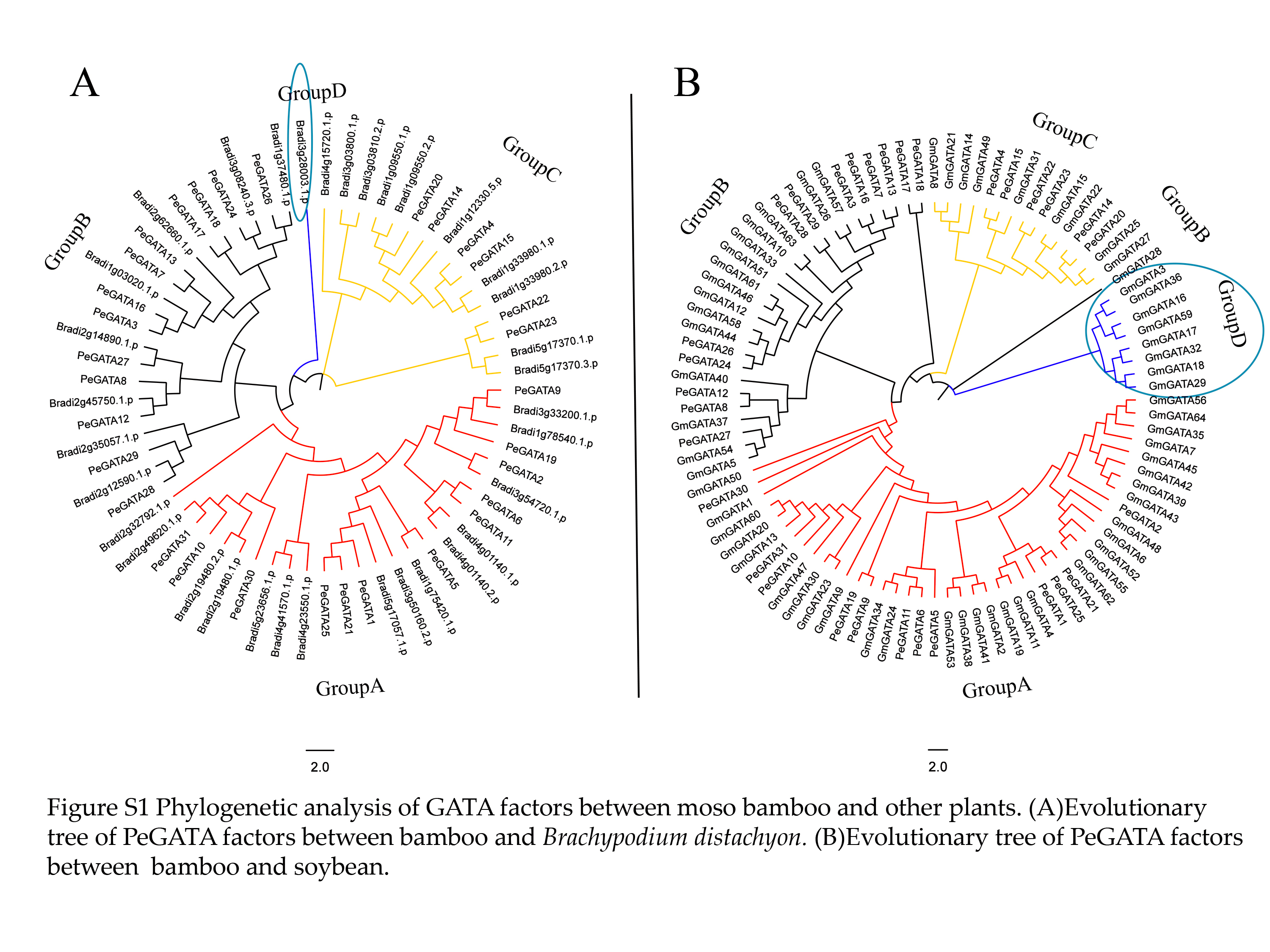

Supplement: Supplementary file 1 [file ijms-21-00014-s001.zip › Fig S1.jpg]
